# Development and validation of a predictive model for double-J stent encrustation after upper urinary tract calculi surgery

**DOI:** 10.1007/s00240-024-01554-7

**Published:** 2024-07-05

**Authors:** Weihui Jia, Wenyu Chi, Chen Liu, Yifen Song, Shunshun Yang, Chonggao Yin

**Affiliations:** 1 Department of Gastroenterology, Weifang People’s Hospital, Shandong, China; 2 Department of Urology, Affiliated Hospital of Shandong Second Medical University, Shandong, China; 3 Nursing College, Shandong Second Medical University, Shandong, China; 4 Department of Pharmacy, Weifang Sunshine Union Hospital, Shandong, China

**Keywords:** Double-J tube, Double-J stent encrustation, Urinary stones, Predictive model, Risk factors

## Abstract

The study is aimed to establish a predictive model of double-J stent encrustation after upper urinary tract calculi surgery. We collected the clinical data of 561 patients with indwelling double-J tubes admitted to a hospital in Shandong Province from January 2019 to December 2020 as the modeling group and 241 cases of indwelling double-J tubes from January 2021 to January 2022 as the verification group. Univariate and binary logistic regression analyses were used to explore risk factors, the risk prediction equation was established, and the receiver operating characteristic (ROC) curve analysis model was used for prediction. In this study, 104 of the 561 patients developed double-J stent encrustation, with an incidence rate of 18.5%. We finally screened out BMI (body mass index) > 23.9 (OR = 1.648), preoperative urine routine white blood cell quantification (OR = 1.149), double-J tube insertion time (OR = 1.566), postoperative water consumption did not reach 2000 ml/d (OR = 8.514), a total of four factors build a risk prediction model. From the ROC curve analysis, the area under the curve (AUC) was 0.844, and the maximum Oden index was 0.579. At this time, the sensitivity was 0.735 and the specificity was 0.844. The research established in this study has a high predictive value for the occurrence of double-J stent encrustation in the double-J tube after upper urinary tract stone surgery, which provides a basis for the prevention and treatment of double-J stent encrustation.

## Introduction

Double-J tube (ureteral stent tube) has been widely used in ureteral obstructions such as urinary stones, urinary system tumors, and ureteral stenosis. It can play an essential role in draining urine and supporting the urethra [1–3]. Upper urinary tract stones include renal stones and ureteral stones, accounting for more than 95% [4]. Double-J stent encrustation formation is one of the most common, underestimated, and less clinically studied complications of indwelling double-J catheters [5, 6]. The formation of double-J stent encrustation may lead to difficult extubation in mild cases and may lead to severe complications such as ureteral rupture and intimal stripping [7–9]. Studies have pointed out that the risk factors for double-J stent encrustation are poor patient follow-up compliance, long indwelling time, co-infection, pyelonephritis, recurrent or residual stone formation, history of stone formation, chronic renal failure, pregnancy metabolism, and congenital abnormalities [10–12].Currently, there is no plan for completely reversing double-J stent encrustation at home and abroad. Most of the research is limited to empirical summary or retrospective research. The clinical countermeasures are mainly symptomatic treatment after double-J stent encrustation. There is still a lack of early quantitative risk assessment strategies. Therefore, seeking a simple and easy-to-use tool to assess postoperative double-J stent encrustation risk is a practical choice to identify high-risk groups and provide adequate clinical intervention support, which is conducive to the most effective use of resources. According to the requirements of “Transparent reporting of a multivariable prediction model for individual prognosis or diagnosis (TRIPOD): the TRIPOD statement,” an international normative guideline for predictive models, an excellent clinical prediction model needs to be externally verified by sample data other than modeling and can be extrapolated sex [13]. Therefore, in this study, the clinical data of 802 patients with double-J stent encrustation were retrospectively collected. A prediction model for double-J stent encrustation was constructed and verified to provide a theoretical and decision-making basis for indwelling double-J tubes to prevent the occurrence of double-J stent encrustation.

## Materials and methods

### Patients

A total of 802 patients with indwelling double-J tubes admitted to a tertiary A hospital in Shandong Province from January 2019 to January 2022 were selected as the research objects. We randomly divided the research subjects into the modeling and verification groups according to the ratio of 7:3, with 561 patients in the modeling group and 241 patients in the verification group. The inclusion criteria were: ①age ≥ 18 years old; ②patients with double-J tube indwelling after upper urinary tract stone surgery. Exclusion criteria: ① lack of complete medical records; ② patients who did not undergo double-J tube extraction in our hospital; ③ patients with ureteral stenosis and other severe urinary system diseases. These patients signed informed consent forms. This study was approved by the Ethics Committee of the Affiliated Hospital of Weifang Medical College (wyfy-2022-ky-051).

### Methods

Based on literature analysis and Delphi expert letters, ten experts who have engaged in clinical work in urology for more than 10 years and have rich clinical experience were selected. We set up our clinical data questionnaire according to experts’ opinions, which includes 18 risk predictors in total. (1) General data of patients: gender, age, genetic disease history, BMI, diabetes, hypertension, preoperative urine routine white blood cell quantification, preoperative urine routine red blood cell quantification, preoperative urine PH; (2) ureteral stent status: double-J tube model, double-J tube brand, double-J tube insertion time, double-J tube insertion times, the number of double-J tubes placed; (3) surgical-related indicators: anesthesia method, surgical method, and operation duration; (4) follow-up factors: daily water intake after an operation.

For stone composition analysis, this study used an infrared spectrometer (SUN-3G type). Place the stone specimen in an oven at 100 °C and heat for 5–10 min to dry. Take a small grain-sized stone specimen and put it in an agate mortar, add dry potassium bromide powder, grind evenly, put the powder into a tableting mold, and use a tablet press at 16 Mpa for 5 s to obtain a uniform translucent tablet, place it in an interpretation analyzer to collect spectra, use computer software to analyze the spectra, and generate a stone report.

### Statistical analysis

We used SPSS 23.0 and R 4.1.2 software for statistical analysis. Normally distributed continuous data were described by mean and standard deviation. An independent *t* test was used for intergroup comparison. The discrete data were described by frequency, composition ratio, or percentage, and the Chi-square and Mann–Whitney *U* tests were used. A logistic regression model was constructed, with double-J stent encrustation as an independent result. Stepwise logistic regression was applied to the factors with a single-factor logistic regression *P* value < 0.10 to screen for double-J stent encrustation risk factors. Patient data were substituted into the logistic regression equation to calculate the predicted probability of double-J stent encrustation for each patient in the verification library. The receiver operating characteristic (ROC) curve was used to predict probability. Furthermore, the bootstrap method was used for internal verification. According to the predicted probability of double-J stent encrustation and the actual occurrence of double-J stent encrustation, the Hosmer–Lemeshow test was used to verify the calibration degree of the established logistic regression model.

## Results

### Patient characteristics

In this study, 104 of the 561 patients in the modeling group developed double-J stent encrustation, with an incidence rate of 18.5%. In 104 patients, the simple stones were mainly composed of anhydrous uric acid 53.85% (56/104) and calcium oxalate monohydrate 10.58 (11/104); mixed stones are mainly calcium oxalate + calcium phosphate 6.74% (7/104). The detailed results are shown in Table 1.

**Table 1:** Analysis of double-J stent encrustation composition and composition ratio

Group	Stone composition	Quantity (*n* = 104)	Proportion. (%)
Simple stones	Anhydrous uric acid	56	53.85
	Calcium oxalate monohydrate	11	10.58
	Calcium oxalate dihydrate	4	3.85
	Amorphous calcium phosphate	2	1.92
	carbonate apatite	2	1.92
	Ammonium hydrogen urate	1	0.96
	Uric acid dihydrate	1	0.96
Mixed stones	Calcium oxalate + calcium phosphate	7	6.86
	Uric acid + sodium urate + calcium oxalate	4	3.85
	Calcium oxalate monohydrate + calcium oxalate dihydrate	3	2.94
	Magnesium ammonium phosphate hexahydrate + calcium carbonate decaoxalate	3	2.94
	Magnesium ammonium phosphate hexahydrate + carbonate apatite	2	1.92
	Magnesium ammonium phosphate hexahydrate + ammonium biurate + apatite carbonate	2	1.92
	Uric acid + sodium urate + calcium phosphate	2	1.92
	Uric acid + sodium urate + calcium oxalate + calcium phosphate	2	1.92
	Ammonium hydrogen urate + anhydrous uric acid	1	0.96
	Ammonium hydrogen urate + calcium phosphate	1	0.96

### Predictive model for double-J stent encrustation

The results of the univariate analysis are listed in Table 2. The results of multivariate analysis showed that BMI > 23.9 (*P* = 0.047), preoperative urine white blood cell quantification (*P* < 0.001), double-J tube insertion time (*P* < 0.001), preoperative daily water intake (*P* = 0.017) are independent risk factors for surface stones in patients (Table 3). According to the logistic regression analysis results, the final formula was obtained as: *Z* = 6.391 + 0.499 × BMI > 23.9 + 0.139 × preoperative urine white blood cell quantification-0.570 × double-J tube insertion time + 2.142 × postoperative daily water intake.

**Table 2:** Univariate analysis of s double-J stent encrustation in double-J tubes

	None (*n* = 457)	Yes (*n* = 104)	*χ*^2^/*Z*	*P*
Gender				
Male	260 (56.9)	65 (62.5)	*χ*^2^ = 1.093	*P* = 0.296
Female	197 (43.1)	39 (37.5)		
Age				
< 25	49 (10.7)	22 (21.2)	*χ*^2^ = 11.280	*P* = 0.004
25–60	374 (81.8)	70 (67.3)		
≥ 60	34 (7.5)	12 (11.5)		
Genetic disease history				
Yes	15 (3.3)	8 (7.7)	*χ*^2^ = 4.191	*P* = 0.041
None				
BMI				
< 18.5	122 (26.7)	27 (26.0)	*χ*^2^ = 82.834	*P* < 0.001
18.5 ~ 23.9	319 (69.8)	45 (43.3)		
> 23.9	16 (3.5)	32 (30.7)		
Diabetes				
Yes	42 (9.2)	24 (23.1)	*χ*^2^ = 15.738	*P* < 0.001
None				
Hypertension				
Yes	41 (9.0)	10 (9.6)	*χ*^2^ = 0.042	*P* = 0.837
None				
Preoperative urine routine white blood cell quantification(u L)	14 (7, 24)	92 (71, 156)	*Z* = − 15.538	*P* < 0.001
Preoperative urine routine red blood cell quantification(u L)	60 (34, 164)	213 (125, 403)	*Z* = − 9.042	*P* < 0.001
Preoperative urine PH	5.4 (5.1, 6.3)	5.6 (5.1, 6.3)	*Z* = − 0.707	*P* = 0.480
Double-J tube model				
4.8F	364 (79.6)	81 (77.9)	*χ*^2^ = 0.161	*P* = 0.688
6F	93 (20.4)	23 (22.1)		
Double-J tube brand				
Jinan Kate	357 (78.1)	88 (84.6)	*χ*^2^ = 2.180	*P* = 0.140
COOK	100 (21.9)	16 (15.4)		
Double-J tube insertion time (d)	29 (23, 34)	39 (28, 46)	*Z* = − 7.525	*P* < 0.001
Double-J tube insertion times				
1 time	419 (91.7)	90 (86.5)	*χ*^2^ = 2.964	*P* = 0.227
2 times	32 (7.0)	11 (10.6)		
3 times	6 (1.3)	3 (2.9)		
The number of double-J tubes placed				
One side	439 (96.1)	97 (93.3)	*χ*^2^ = 1.551	*P* = 0.213
Bilateral	18 (3.9)	7 (6.7)		
Anesthesia method			*χ*^2^ = 1.829	*P* = 0.401
Intravenous general anesthesia	372 (81.4)	88 (84.6)	*χ*^2^ = 0.593	*P* = 0.441
Local infiltration anesthesia	85 (18.6)	16 (15.4)		
Surgical method				
Rigid ureteroscopy lithotripsy	289 (63.3)	65 (62.5)	*χ*^2^ = 0.074	*P* = 0.963
Percutaneous nephrolithotomy	87 (19.0)	21 (20.2)		
Ureteroscopic lithotripsy	81 (17.7)	18 (17.3)		
Operation duration (min)	47 (40, 73)	47 (39, 69)	*Z* = − 0.0.555	*P* = 0.579
Daily water intake after the operation				
Below 2000 ml/d	74 (16.2)	51 (49.0)	*χ*^2^ = 52.781	*P* < 0.001
Up to 2000 ml/d	383 (87.8)	53 (51.0)

**Table 3:** Multivariate analysis of double-J stent encrustation in double-J tubes

Variable	*β*	SE	Wald *χ*^2^	*P*	OR	95% *CI*
Gender	3.153	2.113	2.226	0.136	13.407	0.372
Genetic disease history	0.499	1.320	0.143	0.705	1.684	0.124
BMI > 23.9	0.499	1.32	0.143	0.047	1.648	0.124–7.92
Diabetes	0.495	1.586	0.997	0.755	0.610	0.027
Abnormal quantitative urine white blood cells before the operation	0.139	0.024	34.607	0.000	1.149	1.97–1.204
Abnormal quantitative urine red blood cells before the operation	0.001	0.002	0.488	0.485	1.001	0.998
The time of double-J tube intubation	0.570	0.159	12.808	0.000	1.566	1.414–1.773
Water intake not reaching 2000 ml/d	2.142	0.901	5.647	0.017	8.514	0.414–0.773
Constant	6.391	3.589	3.172	0.075	596.568	–

**Fig. 1:**
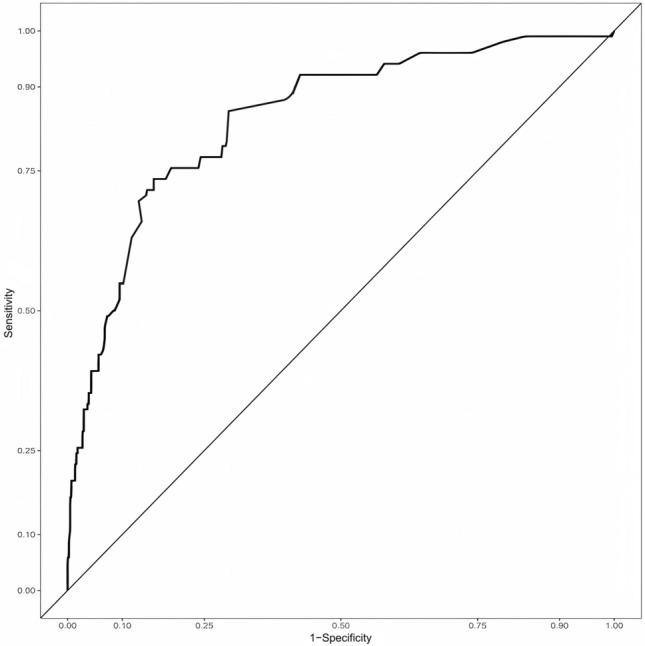
ROC for the prediction model. AUC was 0.844 (95% CI 0.801 ~ 0.887)

**Fig. 2:**
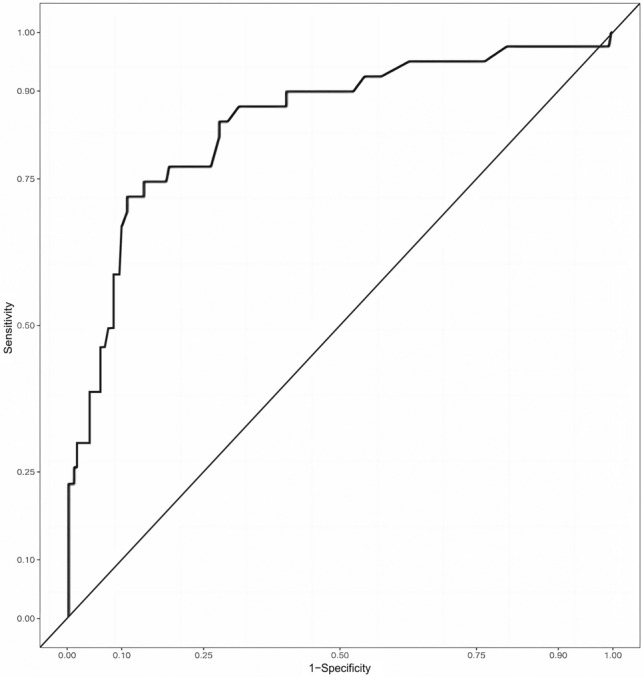
ROC for the prediction model. AUC was 0.854 (95% CI 0.778 ~ 0.929)

The areas under the curve (AUC) of the ROC curves of the modeling group was 0.844 (95% CI 0.801 ~ 0.887), the maximum Oden index was 0.579, the sensitivity at this time was 0.735, and the specificity was 0.844 (Fig. 1). We substituted 241 patients from the validation group into this model, and 41 patients developed double-J stent encrustation, an incidence rate of 17.0%. The areas under the curve (AUC) of the ROC curves of the validation group is 0.854 (95% CI 0.778 ~ 0.929), the maximum Oden index is 0.61, the sensitivity is 0.718, and the specificity is 0.892 (Fig. 2).

## Discussion

In this study, there were four independent factors affecting the occurrence of double-J stent encrustation after upper urinary tract calculi surgery. The four independent factors are BMI > 23.9, preoperative urine routine white blood cell quantification, double-J tube insertion time, postoperative water consumption did not reach 2000 ml/d.

This study found that BMI > 23.9 is a risk factor for double-J stent encrustation (OR = 1.648). Urinary calculus is not an isolated disease but the result of the joint action of metabolic syndrome, obesity, diabetes, and cardiovascular disease. However, obesity is a factor that can be controlled artificially [14]. Common complications of obesity are hypertension and diabetes, which have a bidirectional relationship with stones [15]. A cross-sectional study of 605 participants followed up for an average of 47 months indicated a direct relationship between stone disease and BMI, which can be used as a predictor of stone disease development in clinical practice [16]. Therefore, in clinical work, medical staff should pay attention to patients’ weight management. During discharge education, we should formulate individualized weight loss programs for obese patients, eat a sensible diet, strengthen exercise, and regularly monitor measurements; we should also strengthen discharge follow-up, regularly inquire about the implementation of the weight loss program, improve the patient’s awareness and attention, and effectively control the weight.

It was found that abnormal quantitative urine white blood cells before the operation was a risk factor for double-J stent encrustation (OR = 1.149). Studies have pointed out [5] that the proportion of double-J stent encrustation formation in patients with urinary tract infections (92.9%) is higher than that in non-infected patients (73.2%). This reminds us to be vigilant in clinical work for those with abnormal quantitative urine white blood cells before operation. If there is any abnormality, the medical staff should conduct bacterial culture, identification, and drug sensitivity test in time and use antibiotics reasonably according to the test results for treatment; medical staff rationally use antimicrobial drugs when discharged from the hospital. At the same time, they should instruct patients to strengthen their nutrition, exercise regularly, improve immunity, and implement whole-process health management to improve patient maintenance compliance and reduce the incidence of urinary tract infections.

This study found that the time of double-J tube intubation was a risk factor for double-J stent encrustation (OR = 0.566). Studies have shown that the only risk factor for double-J tube stones is the indwelling time [17]. In one study, a total of 47.0% of stents encrusted, with encrustation rates ranging from 26.8% for less than 6 weeks, 56.9% for 6 to 12 weeks, and 75.9% for more than 12 weeks [18]. Studies by Legrand F et al. have shown that the time of double-J tube intubation is positively correlated with the incidence of double-J stent encrustation [19]. Therefore, medical staff should follow-up in time to remind patients to take the tube on time. Due to the condition requiring a longer indwelling time, the medical staff reminds the patient to recheck regularly and shorten the double-J tube insertion time after understanding the patient’s urinary stones composition and body metabolic environment.

This study found that water intake not reaching 2000 ml/d was an independent risk factor for surface stones (OR = 8.514). Tasian et al. [20] study pointed out that less than 50% of patients ensured adequate daily fluid intake. In a large meta-study [21], compared with those who consumed less than 1000 mL/d, the risk of urinary stones was reduced by about half in those who consumed more than 2000 mL/d(RR = 0.56, 95% CI 0.48–0.65, *P* < 0.001), but there is still some controversy about the intake of liquid types, such as tap water, mineral water, fruit juice, tea, and coffee. Therefore, medical staff should emphasize that the amount of drinking water should be more than 2000 ml/d, and recommend that patients carry drinking water bottles with scales or capacity marks every day to improve patient compliance; at the same time, medical staff should strengthen follow-up visits, adopt live webcasts, and establish WeChat groups Communication and other means to make patients aware of the importance of drinking more water for the prevention of double-J stent encrustation.

## Limitations

This study is a retrospective case–control study, so the data obtained are limited. Double-J stent crust. other risk factors such as patient’s occupation, geographic location, lifestyle, dietary habits, and residual stones were not included. However, rigorous external verification has been carried out, and the collected cases are from the same hospital with a small sample size; the predictive effect of the risk model needs further research with a larger sample size to verify. The following research direction is to improve the model and apply it in clinical practice, carry out multi-center application promotion, further optimize and improve the values in the model, and make it closer to actual clinical application.

## Conclusion

In this study, a prediction model for double-J stent encrustation with indwelling double-J tubes was constructed after upper urinary tract stone surgery. There were four independent factors affecting the occurrence of double-J stent encrustation after upper urinary tract calculi surgery. The four independent factors are BMI > 23.9, preoperative urine routine white blood cell quantification, double-J tube insertion time, postoperative water consumption did not reach 2000 ml/d. It has a good predictive ability. We recommend that clinical staff use this risk prediction model to implement targeted interventions to reduce the probability of double-J stent encrustation.

## Data Availability

All the data for this study are from the information management system of the Weifang People’s Hospital, which is available from the corresponding author on reasonable request.

## Abbreviations

ROC: The receiver operating characteristic
AUC: Area under the receiver operating characteristic curve
TRIPOD: Transparent reporting of a multivariable prediction model for individual prognosis or diagnosis
BMI: Body mass index
